# Distinct patterns and interactions of microbiota and short-chain fatty acids in breast milk and infant gut from rural and urban dyads

**DOI:** 10.3389/fmicb.2026.1814630

**Published:** 2026-05-29

**Authors:** Saeid Khakisahneh, Eunseo Lee, Amy Hui, Ruozhi Zhao, Fei Huang, Xue-Ying Zhang, Garry X. Shen

**Affiliations:** 1 Department of Internal Medicine, University of Manitoba, Winnipeg, MB, Canada; 2 Department of Food and Human Nutritional Sciences, University of Manitoba, Winnipeg, MB, Canada; 3 State Key Laboratory of Animal Biodiversity Conservation and Integrated Pest Management, Institute of Zoology, Chinese Academy of Sciences, Beijing, China

**Keywords:** breast milk microbiota, infant gut microbiota, microbiota diversity, rural and urban residency, short-chain fatty acids

## Abstract

**Introduction:**

Breastfeeding is crucial for the development of infant gut microbiota and long-term health. The influence of geographical residency on the composition and interactions among milk and gut microbiota, their metabolites, including short-chain fatty acids (SCFAs), remains unclear.

**Methods:**

Infant stool (0-12 months) and breast milk samples (1-30 days postpartum) were collected from 69 dyads living in rural or urban Manitoba, including 10 mothers diagnosed as gestational diabetes, for microbiota and SCFA analyses using 16S rRNA sequencing or gas chromatography–mass spectrometry.

**Results:**

Infant gut microbiota maturity was significantly higher in rural infants during the early infancy (0-2 months) compared to urban infants, but urban infants surpassed their rural counterparts during late infancy (6-12 months). Mature milk from urban mothers contained higher levels of *Veillonella* and *Alistipes*, and lower isobutyric acid than mature milk from rural mothers. A significant correlation of *Alistipes* between milk and infant stool was detected in urban dyads, and milk-gut sharing of *Blautia* was observed in rural dyads. Rural infants exhibited higher levels of fecal *Faecalibacterium*, *Odoribacter*, and acetic acid. Urban infant stool contained higher levels of *Bacteroides*, *Akkermansia*, *Ruminococcaceae*, and valeric acid.

**Discussion:**

The gut microbiota of rural infants showed unstable microbial succession, while urban infants displayed strong gut-gut continuity, suggesting stable maturation and potential health benefits. The results of the present study revealed distinct patterns and interactions of microbiota and SCFAs in breast milk and infants’ stool from rural versus urban dyads.

## Graphic abstract

**Figure:**
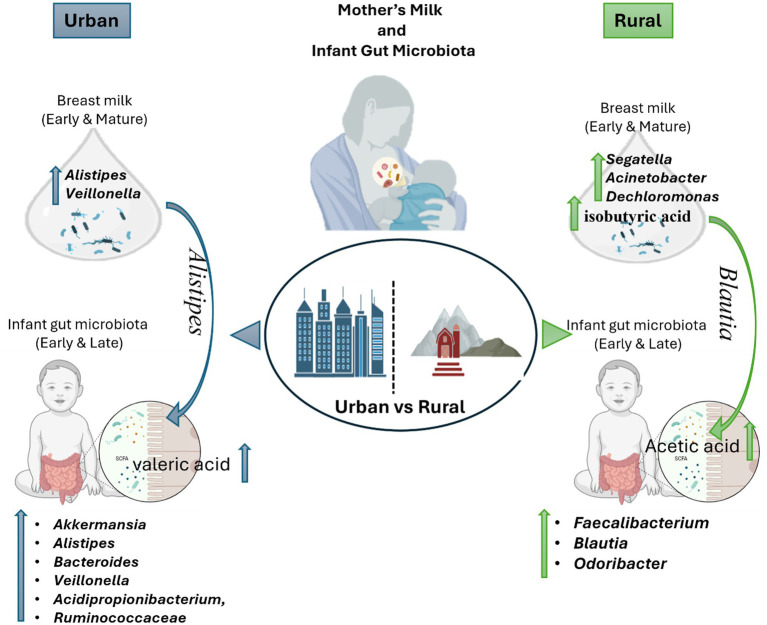
A schematic illustration showing differences between breast milk and infant gut microbiota, as well as short-chain fatty acids (SCFA), in urban and rural residents.

## Highlights

- Rural mature milk had a higher level of isobutyric acid than urban milk.
- Urban mature milk had higher abundances of *Veillonella* and *Alistipes*.
- Milk-gut sharing of *Alistipes* in urban and *Blaucia* in rural dyads was detected.
- Fecal *Faecalibacterium*, *Odoribacter*, and acetic acid were higher in rural infants.
- Fecal *Bacteroides*, *Akkermansia*, and valeric acid were higher in urban infants.

## 1 Introduction

The development of gut microbiota in infants starts at birth (Milani et al., 2017). Maternal milk microbiota is a key source of infants’ gut microbiota, which plays a central role in the metabolism and immune response of infants (Fehr et al., 2020; Ma et al., 2022). Breast milk is recognized as the best food for infants, and it is a dynamic, complex biological fluid containing small amounts of bacteria. These bacteria include microbes from maternal skin and/or from the infant’s oral cavity, which travel retrogradely into maternal milk ducts, or originate from other endogenous or environmental sources (Fehr et al., 2020; Moossavi et al., 2019). Short-chain fatty acids (SCFAs) have also been found in breast milk, but their source remains uncertain. Since SCFA levels in milk are linked to SCFA-producing microbes in milk, SCFAs in milk may come from the digestion of carbohydrates in milk by bacteria (Xi et al., 2024). Milk bacteria and SCFAs can directly enter the infant’s gut and influence the development of gut microbiota, metabolism, and immunity (Shenhav et al., 2024; Xi et al., 2024). Milk production begins a few weeks before delivery, known as colostrum, which contains abundant proteins, antibodies, and cells. The secretion of colostrum usually stops within days after birth. The secretion of mature milk increases subsequently, which includes fewer proteins, antibodies, and cells than colostrum (Moltó-Puigmartí et al., 2011). However, limited knowledge is available about the interactions between early and mature milk microbiota and SCFAs (Fehr et al., 2020; Ma et al., 2022).

Rural and urban disparities significantly influence health outcomes. According to Statistics Canada, rural residents were 1.5 times more likely to develop diabetes than their urban counterparts in Canada. Major contributors to this healthy burden for rural residents in Canada were identified as inadequate access to health care and the educational system, and less access to healthy foods and physical inactivity (Spark conferance, 2025). Breastfeeding initiation is a negative modulator of postpartum diabetes in mothers and youth-onset type 2 diabetes (T2D) in offspring in Manitoba (Martens et al., 2016). The rate of breastfeeding initiation in rural, including remote and regions in Manitoba during 2014–2021 was 79.1%, which was significantly lower than that in urban regions in the province (86.1%, *p* < 0.0001) (Hui et al., 2025). Geographical residency is widely recognized as a critical compounding factor influencing the health of pregnancy and infants, serving as a driver for a wide range of environmental, cultural, and lifestyle variations. The exposure and colonization of microbiota in breast milk can vary significantly between rural and urban settings, affected by sanitation, water quality, diet, housing, antibiotic exposure, breastfeeding practices, weather, culture, ethnicity, and socioeconomic differences (Morandini et al., 2023; Xie et al., 2022). The impact of geographic residency on the microbiota in breastmilk and its relationship with infant gut microbiota in Manitoba remains unclear.

SCFAs are produced from fiber fermentation in the gut by a large community of bacteria, which are essential for maintaining intestinal balance, strengthening the epithelial barrier, and regulating metabolic and immune responses systemically (Ma et al., 2022; Wang S. et al., 2025). For instance, T2D has been linked to gut dysbiosis and lower SCFAs levels in both children and adults (Wang S. et al., 2025; Wang Y. et al., 2025). Breast milk contains SCFAs, which are expected to enter the gut of infants. Analyzing the composition of SCFAs in parallel to microbiota in breast milk and infants’ feces living in urban versus rural regions may offer mechanistic insights into the succession and interactions between bacteria and SCFAs in maternal milk and infants’ gut, and the relationship between geographic residency-associated discrepancies and metabolic health in rural versus urban residents.

The present study examined microbiota and SCFAs in the breast milk of mothers and in the stool of their offspring living in rural and urban regions of Manitoba. The impact of geographic residency on the composition and succession of bacteria and SCFAs in early versus mature breast milk and early versus late infants’ gut was further investigated.

## 2 Methods

### 2.1 Study population and design

Sixty-nine pregnant women living in Manitoba, Canada, were recruited from rural areas (*n* = 29) or from urban Winnipeg (*n* = 40). Inclusion criteria: (i) pregnant women delivered infants in Manitoba; (ii) agree to submit infant stool samples and breast milk samples as scheduled; (iii) sign an informed consent. Exclusion criteria: (i) women with pre-pregnancy diabetes; (ii) mothers received antibiotic or commercial probiotic therapy during within 1 month around labor; (iii) sample excluded from analysis for infants received antibiotic therapy within one month of stool sample collection. All infants were singletons at birth. Sixteen infants were delivered by cesarean section (C-section) and 63 were naturally delivered via virginal route. Ten participants, 9 from urban, 1 from rural, were diagnosed with gestational diabetes mellitus (GDM) (Table 1). None of participants had known pre-pregnancy diabetes. The diagnosis of gestational diabetes mellitus (GDM) was using a two-step method as described in Clinical Guidelines of Diabetes Canada. Briefly, all pregnant women in Canada are required to have a GDM screening using a non-fasting 50 g oral glucose challenge test between 24–28 weeks of pregnancy. For individual whose 1 h postprandial plasma glucose ≥11.1 mM/L, diagnosis of GDM was confirmed and no further test required. For those with postprandial plasma glucose between 7.8 and 11.0 mM/L, 75 g glucose tolerance test (OGTT) was conducted. If fasting plasma glucose ≥5.3, or 1 h or 2 h post prandial plasma glucose ≥10.0 or 9.6 mm/L, the diagnosis of GDM was confirmed (Diabetes Canada Clinical Practice Guidelines Expert Committee, 2018). Infant feeding status was recorded during home visits and categorized as exclusive breastfeeding feeding (EBF), mixed feeding (partial breastfeeding mixed with formula feeding) and formula-feeding (For details of infant feeding in individual participants, see Supplementary Table S1). Solid foods were introduced in most of infants beyond 6 months of age.

**Table 1:** Summary of demographic characters of participants.

Residential region	Total number (*n*/%)	Virginal delivery (*n*/%)	C-section delivery (*n*/%)	Exclusive breastfeeding (*n*/%)	Mixed feeding (*n*/%)	Formula feeding (*n*/%)	Gestational diabetes (*n*/%)
Urban	40 (58.0)	30 (75.0)	10 (25.0)	30 (75.0)	8 (20.0)	2 (5.0)	9 (22.5)
Rural	29 (42.0)	23 (79.3)	6 (20.7)	19 (65.5)	7 (24.1)	3 (10.3)	1 (3.4)

### 2.2 Ethical statement

Experimental protocols and consent form were approved by Human Research Ethics Board at the University of Manitoba (identification code HS21031) on September 18, 2017. All participants signed informed consent for the project. All experimental methods were carried out in accordance with relevant ethical guidelines and regulations.

### 2.3 Sample size justification

The present study as a pilot prospective cohort did not perform a formal power calculation. The sample size of the cohort was estimated based on sample sizes (total *n* = 23–30, 13-16/group) of previous studies in literature with comparable designs and similar methods (Du et al., 2025, Li et al., 2022).

### 2.4 Sample collection

Sample collections started in 2018 and ended in 2025. Participants were instructed to manually collect 0.5–1.5 mL of breast milk after nipples were wiped with sterile non-alcohol wet tissue without assistance from a pump at 1–14 days (early) and 20–30 days (mature) postpartum. Breast milk was dripped in sterile cup and then pulled into sterile tubes prefilled with milk preservation solution E (Cat. 90,095, Norgen Biotek, Thorold, ON, Canada). The samples were kept at room temperature for less than one month, as instructed by the manufacturer (Norgen Biotek). Milk samples were collected at same day per time point. Infant stool (0.5–1 g) was collected from diaper with sterilized wiper at 0–2 months (early infancy) and 6–12 months (late infancy) in stool nucleic acid collection and preservation tubes (Cat. 45,660, Norgen) and kept at room temperature for <12 months as instructed. No specific dietary requirement for mothers or infants was requested during sample collection. Samples were aliquoted by laboratory staff within the instructed period and stored at −70 °C until shipment with dry ice for analysis.

### 2.5 DNA extraction, 16S rRNA gene amplicon sequencing

The Milk DNA Preservation and Isolation Kit (Norgen, Cat. 44,800) was used to extract DNA from breast milk, including a brief centrifugation to remove lipids. Lysozyme and proteinase K were applied for enzymatic processing following the manufacturer’s protocol. DNA concentration and purity were measured with a Nanodrop spectrophotometer.

Stool DNAs were extracted using the QIAamp PowerFecal DNA Kit (QIAGEN, Denmark) and prepared for 16S rDNA sequencing at the Integrated Microbiome Resource (IMR) at Dalhousie University. Primers 515F (5’-GTGYCAGCMGCCGCGGTAA-3′) and 926R (5’-CCGYCAATTYMTTTRAGTTT-3′) were used to amplify bacterial genes in the V4–V5 region of the 16S rRNA gene via polymerase chain reaction, and the Just-a-Plate 96-well kit (Charm Biotech, Cape Girardeau, MO) was used for amplicon normalization. Sequencing was performed on an Illumina MiSeq platform (San Diego, CA) at IMR. To process demultiplexed reads, Cutadapt and DADA2, implemented in QIIME2, were used for primer removal and sequence denoising, respectively (Bolyen et al., 2019; Callahan et al., 2016; Martin, 2011). A RESCRIPt-trained classifier based on the SILVA 138.2 database was used to assign taxonomy (Quast et al., 2013). For quality control, bacterial taxa accounting for less than 0.1% of the total read count were filtered out—sequences with fewer than 400 reads for milk and fewer than 2,000 reads for stool per sample were excluded. Data were normalized using total sum scaling to relative abundance or scaled to the median library size across samples. DNA sequences of 150 milk samples were obtained, including 46 mature milk (20–30 days postpartum) and 104 early milks (0–14 days postpartum). Sequences were obtained from 146 infant stool samples, including 96 during the early stage (0–2 months) and 50 during the later stage (6–12 months, Supplementary Tables S2, S3).

### 2.6 Short-chain fatty acids (SCFAs)

Milk and stool samples were derivatized using propyl chloroformate, water, propanol, and pyridine. SCFAs were analyzed at Microbiome Insights (Vancouver, BC) with an Agilent 7890A gas chromatography coupled to a 5975A mass spectrometry detector (Zheng et al., 2013).

In brief, SCFAs in breast milk and stool samples were measured as absolute concentrations (mM) using external calibration curves created from genuine SCFA standards. To account for analytical variability, concentrations were normalized relative to the internal standard (2-ethylbutyric acid). Because the matrices were different, breast milk samples had to go through acidification followed by centrifugation and careful collection of the aqueous phase while avoiding the lipid layer, after being acidified and centrifuged. On the other hand, stool samples had to be mechanically homogenized before extraction to make sure the matrix was completely broken up. Quantification of SCFAs was performed using Chromeleon chromatography software (Thermo Fisher Scientific), and the limit of detection for individual SCFAs under the described gas chromatography Flame Ionization Detector conditions was approximately 0.15625 mM. The limit of quantification (LOQ) was defined as the lowest calibration point meeting acceptable linearity and signal-to-noise criteria.

Only samples that met quality control standards, such as detectable internal standard recovery, correct retention time matching, and peak intensities above LOQ, were considered in further analyses, indicating that only samples meeting predefined analytical qualification criteria were included, which include 140 stool SCFAs and 141 milk SCFAs.

### 2.7 Statistical analysis

All statistical analyses were performed using R Studio (v4.3.3). Phyloseq objects were created from amplicon sequence variant (ASV) tables. Bray-Curtis distances were used to assess microbiota composition (*β*-diversity), and the vegan package was utilized for distance-based redundancy analysis (dbRDA). Several 9,999 permutations were conducted to determine statistical significance (Oksanen et al., 2022). Heatmaps displayed taxa present in at least 30% of samples with a minimum of 1% relative abundance (Lahti et al., 2014; Shade et al., 2013). Genus’s level relative abundances within the early and late phases were compared between the urban and rural groups separately by using the Wilcoxon rank-sum test. It documented that if we considering consistency and sensitivity together for analyzing relative abundances, this nonparametric method was the best performance (Pelto et al., 2025; Tong et al., 2020). Only taxa present in both groups were included in the analysis following prevalence and abundance filtering. The rank-based statistic (r) was used to estimate the effect size, and the corresponding R^2^ value was computed to measure the percentage of variance explained.

All regression analyses were adjusted for potential confounders, including batch, GDM, breastfeeding, and delivery method. Linear mixed-effects models (LMMs) were used to analyze alpha diversity indices, SCFA concentrations, and microbiota maturity scores. In these models, geographic residency (urban versus rural), lactation stage (early versus late), and their interaction were included as fixed effects, along with batch, GDM status, breastfeeding mode, and delivery method as covariates. A random intercept for SubjectID was included to account for repeated measures within individuals. The model structure was specified as:

$\begin{array}{l} \mathrm{Yij}=\beta 0+\beta 1\mathrm{typ}2\mathrm{ij}+\beta 2\text{Ethnicityij}+\beta 3\text{Deliveryij}\\ +\beta 4\text{Batchij}+\mathrm{ui}+\mathrm{\epsilon ij} \end{array}$

In the model, $Y_{\mathrm{ij}}$ represents measurement for subject *i* at time point *j*. $\beta_{0}$ represents the overall intercept. The β coefficients represent the effects of lactation stage, ethnicity, delivery mode, and batch. A random intercept ( $u_{i}$ ) was included for each subject to account for repeated measurements from the same individual. The residual error term ( $\epsilon_{\mathrm{ij}}$ ) represents unexplained variation after accounting for the fixed and random effects.

Partial Spearman rank correlation was employed to examine relationships between microbial taxa and SCFA concentrations after the adjustment of relevant confounders. Alpha diversity, SCFA concentrations and microbiota maturity scores were analyzed using LMM. The Benjamini–Hochberg false discovery rate adjustment was applied to *p*-values to account for multiple testing in differential abundance and correlation analyses. The NetCoMi package was used to analyze bacterial co-occurrence networks. The most influential microorganisms within the network were identified based on their eigenvector centrality scores. Hub nodes with a centrality value above the empirical 95% quantile were considered, and their sizes were determined using eigenvector centrality (Peschel et al., 2021).

## 3 Results

### 3.1 Geographical residency and lactation stages affect the composition of milk microbiota

**Figure 1:**
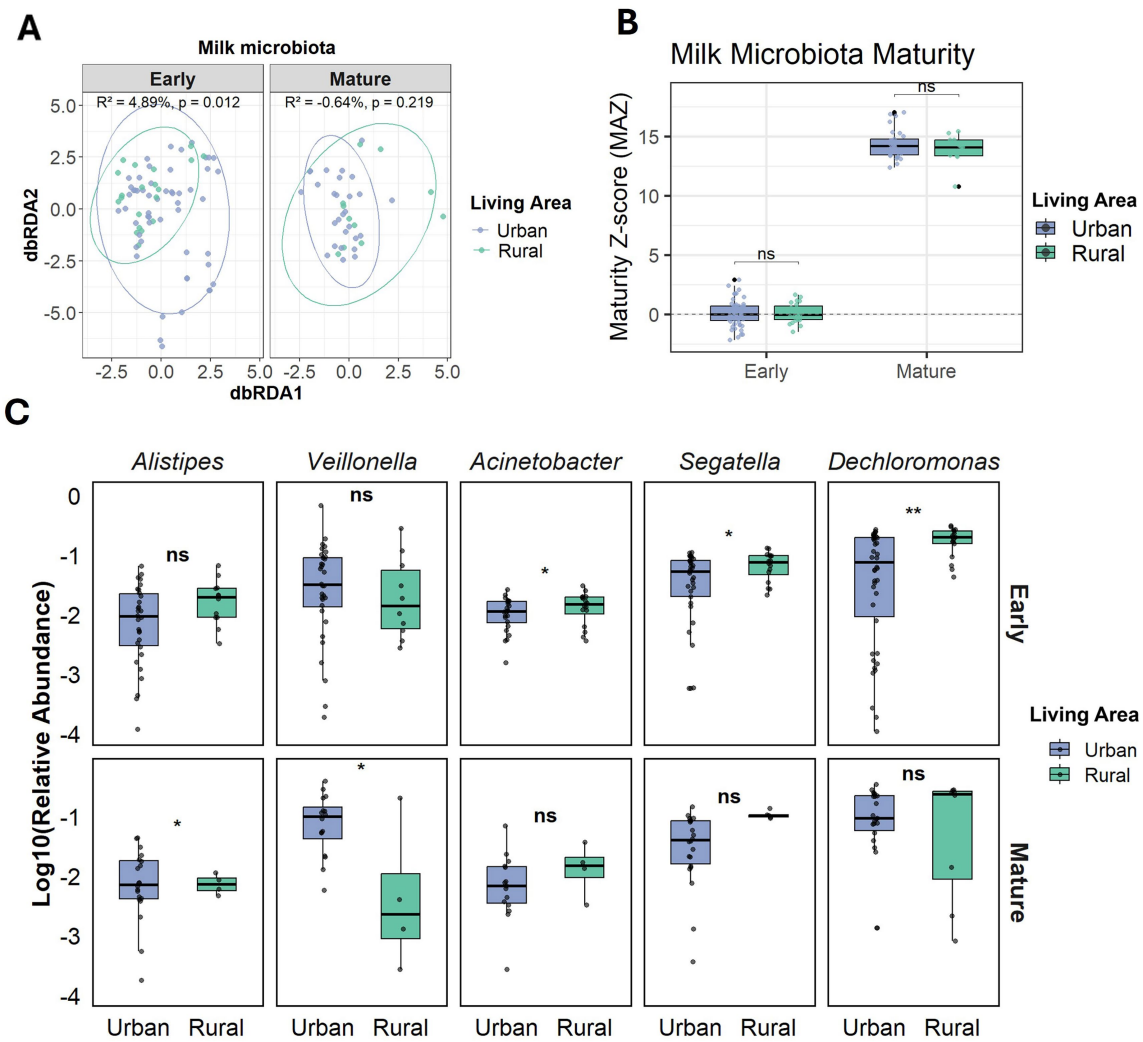
Differences between urban and rural areas in the diversity and composition of the human milk microbiota at the early and mature stages of lactation. **(A)** Distance-based redundancy analysis (dbRDA) of genus-level profiles comparing urban and rural milk microbiota for early and mature milk. Ellipses indicate 95% confidence intervals. R^2^ and *p*-values are shown. **(B)** Comparison of milk microbiota maturation patterns between rural and urban areas. ns, not significant. **(C)** Boxplots of log10-transformed relative abundance of selected genera at early and mature milk. *p (FDR) < 0.05; **p (FDR) < 0.01.

Notable differences in milk composition between urban and rural groups were observed in early milk (1–14 days, R^2^ = 2.41%, *p* = 0.014) but not in mature milk (20–30 days, R^2^ = 0.1%, *p* = 0.126), based on *β*-diversity analysis (Figure 1A). The pattern of milk maturation assessed with microbiota-by-age z-scores (MAZ) did not show a significant difference between urban and rural groups (Figure 1B). The boxplots of relative abundances of phylum taxa demonstrated significantly higher abundances of *Cyanobacteria* and *Pseudomonadota* in rural early milk, but significantly higher *Bacillota* and *Bacteroidota* in rural mature milk compared to corresponding urban milk (Supplementary Figure S1). The boxplots of relative abundances of genus taxa in milk showed that rural samples had significantly more *Acinetobacter*, *Segatella*, and *Dechloromonas* in early milk. Urban mature milk showed significantly higher levels of *Veillonella* and *Alistipes* compared to rural counterparts, but no significant difference was detected between urban and rural early milk (Figure 1C).

### 3.2 Geographic-specific differences in breast milk SCFA_S_

**Figure 2:**
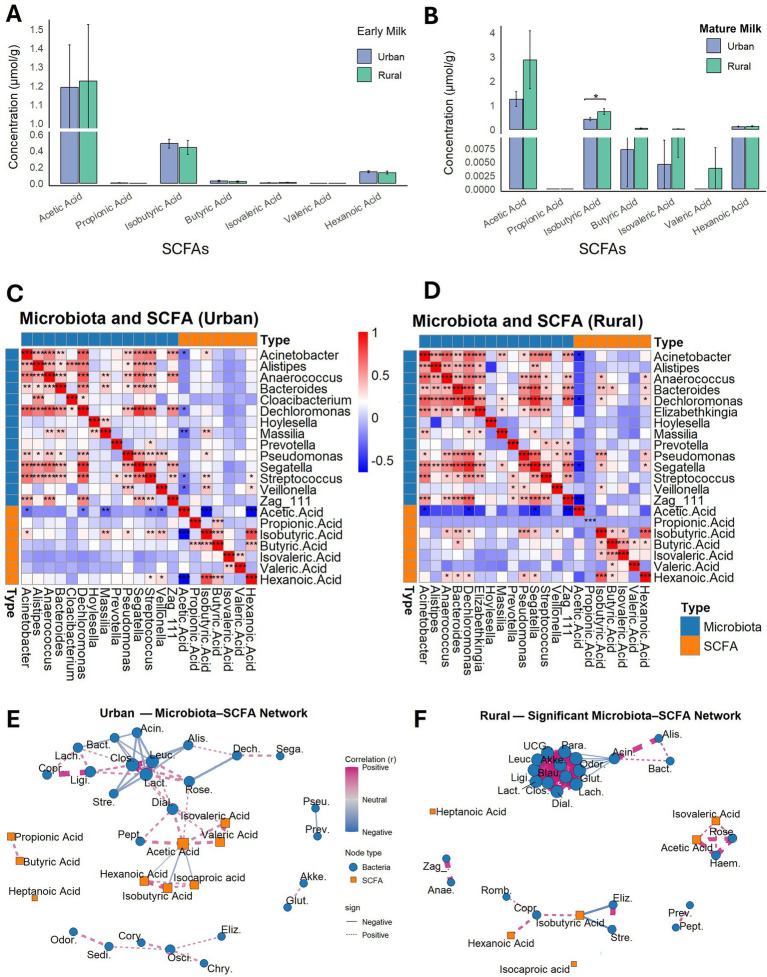
Interactions between urban–rural microbial and milk short-chain fatty acids (SCFAs) in early and mature breast milk stages. Normalized concentrations of SCFA in breast milk from urban and rural mothers during **(A)** early and **(B)** mature stages. Asterisk denotes statistical significance (Wilcoxon test, *p* (FDR) < 0.01; ns, not significant) between urban and rural milk. Spearman correlation heatmaps between relative abundances of major milk bacterial genera and SCFAs concentrations for **(C)** urban and **(D)** rural milk samples. Red and blue indicate positive and negative correlations, respectively. Co-occurrence networks between milk bacteria genera and SCFAs in **(E)** urban and **(F)** rural early infant stool samples *p (FDR) < 0.05; **p (FDR) < 0.01; ***p (FDR) < 0.001. Early milk: 0–14 days; mature milk: 15–30 days. Acin., *Acinetobacter*; Acut., *Acutalibacter*; Akke., *Akkermansia*; Alis., *Alistipes*; Anae., *Anaerococcus*; Bact., *Bacteroides*; Blau., *Blautia*; Chry., *Chryseobacterium*; Coll., *Collinsella*; Cory., *Corynebacterium*; Dial., *Dialister*; Eliz., *Elizabethkingia*; Faec., *Faecalibacterium*; Glut., *Glutamicibacter*; Haem., *Haemophilus*; Hoyl., *Hoylesella*; Inte., *Intestinibacter*; Lach., *Lachnospiraceae_UC008*; Lact., *Lactobacillus*; Leuc., *Leuconostoc*; Ligi., *Ligilactobacillus*; Meth., *Methylobacterium*; Meth.*2*, *Methylomusa; Odor.*, *Odoribacter; Osci.*, *Oscillibacter; Para.*, *Parabacteroides; Pept.*, *Peptoniphilus; Prev.*, *Prevotella; Pseu.*, *Pseudomonas; Romb.*, *Romboutsia; Rose.*, *Roseburia; Ruth.*, *Ruthenibacterium; Sali.*, *Salinispira; Sedi.*, *Sediminibacterium; Thom.*, *Thomasclavelia; Turi.*, *Turicibacter; Stre.*, *Streptococcus; UC.*, *UC002; Zag*., *Zag_111*.

Levels of isobutyric acid in mature milk from urban mothers were significantly lower than those from rural mothers (*p* < 0.01). No significant differences were observed in other SCFAs in mature or early milk (Figures 2A,B). Notably, *Pseudomonas* and *Veillonella* positively correlated with isobutyric acid in both rural and urban milk, but a positive association between *Segatella*, *and Bacteroides* with isobutyric acid was only observed in rural group (*p* < 0.05 or 0.001; Figures 2C,D). Additionally, in rural areas, hexanoic acid was significantly correlated with *Segatella*, *Anaerococcus*, *Bacteroides*, *Dechloromonas*, and *Pseudomonas* (*p* < 0.05 or 0.01; Figures 2C,D). Co-occurrence network analysis revealed a positive interaction of *Peptoniphilus* and *Dialister* with acetic acid, as well as a negative interaction of acetic acid with isobutyric and hexanoic acids in milk from urban-ling mothers (Figure 2E). In the rural area, mothers’ milk, *Romboutsia*, and *Corynebacterium* positively interacted with isobutyric acid, as well as hexanoic acid showed a positive interaction with isobutyric acid (Figure 2F).

### 3.3 Influence of residential location on infant gut microbiota

**Figure 3:**
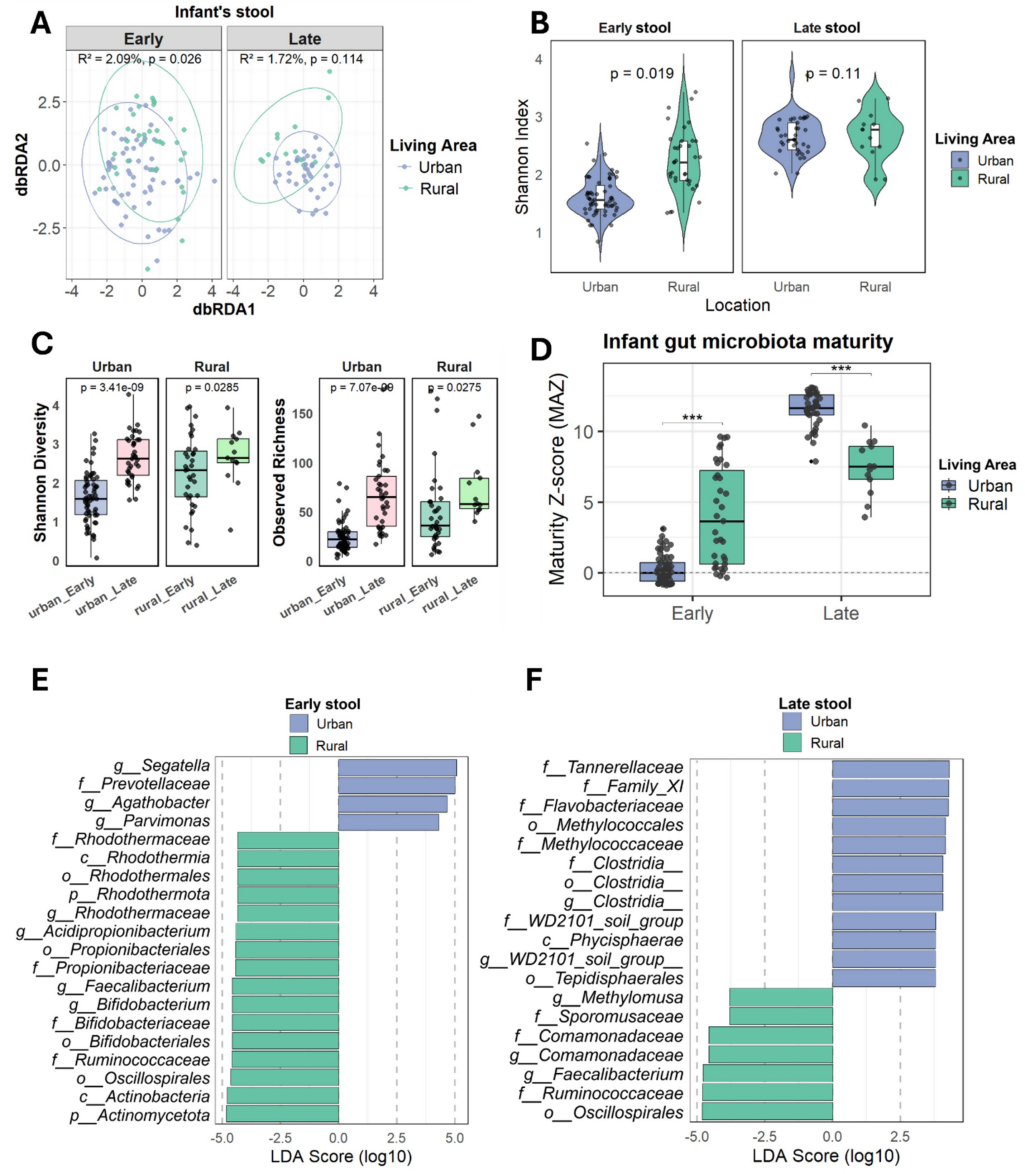
Impact of residing in a rural or urban environment on the diversity of the infant’s gut microbiota and microbial network structure. **(A)** Distance-based redundancy analysis (dbRDA) of genus-level infant gut microbiota profiles at early (0–2 months) and late (6–12 months) stages across urban and rural locations. Ellipses represent 95% confidence intervals. R^2^ and *p*-values are shown. **(B)** Violin plots showing Shannon diversity between groups. **(C)** Boxplots comparing Shannon diversity and observed richness within and between groups. Urban infants showed significantly increased diversity in late vs. early stages. **(D)** Comparison of infant gut microbiota maturation patterns between rural and urban areas. ***p < 0.001. **(E,F)** LDA of taxa enriched in rural vs. urban infant gut microbiota during **(E)** early and **(F)** late infant stool stages. Taxa enriched in rural infants are shown in green; urban-enriched in blue. Bar plots showing normalized concentrations of SCFA between urban and rural areas in early and late stages of infant gut stool. *p < 0.05.

During early infancy (0–2 months), a significant difference in microbial composition was observed between urban and rural infant fecal samples (R^2^ = 2.09%, *p* = 0.026), which was less prominent in later infancy (R^2^ = 1.72%, *p* = 0.114; Figure 3A). The gut microbiota of rural, but not urban, infants showed higher *α*-diversity and richness during early infancy compared to late infancy (Shannon index, *p* = 0.019; Chao1 index = 0.0265) (Figure 3B). Although the Shannon index increased from early to late stages in both groups, the urban cohort demonstrated a notably sharper rise than the rural cohort (*p* < 0.001) (Figure 3C). Infant gut microbiota maturity was higher in rural infants during early infancy, but that of urban infants surpassed rural counterparts during late infancy (*p* < 0.01–0.001, Figure 3D). Linear discriminant analysis (LDA) showed that four distinctive bacterial taxa were present in the urban group and 16 in the rural group during early infancy, and the trend in the number of distinct bacteria between the groups reversed during late infancy. The stool of rural infants enriched by *Faecalibacterium*, *Ruminococcaceae*, and *Oscillospirales* in both early and late infancy. However, most of the other taxa enriched in the early stage failed to dominate during the late stage in urban infants. In contrast, 12 taxa, including *Clostridia* from order to genus level, *Methylcoccales* order bacteria, and *Methylcoccaceae* family bacteria, were enriched in urban late infancy stool (Figures 3E,F).

**Figure 4:**
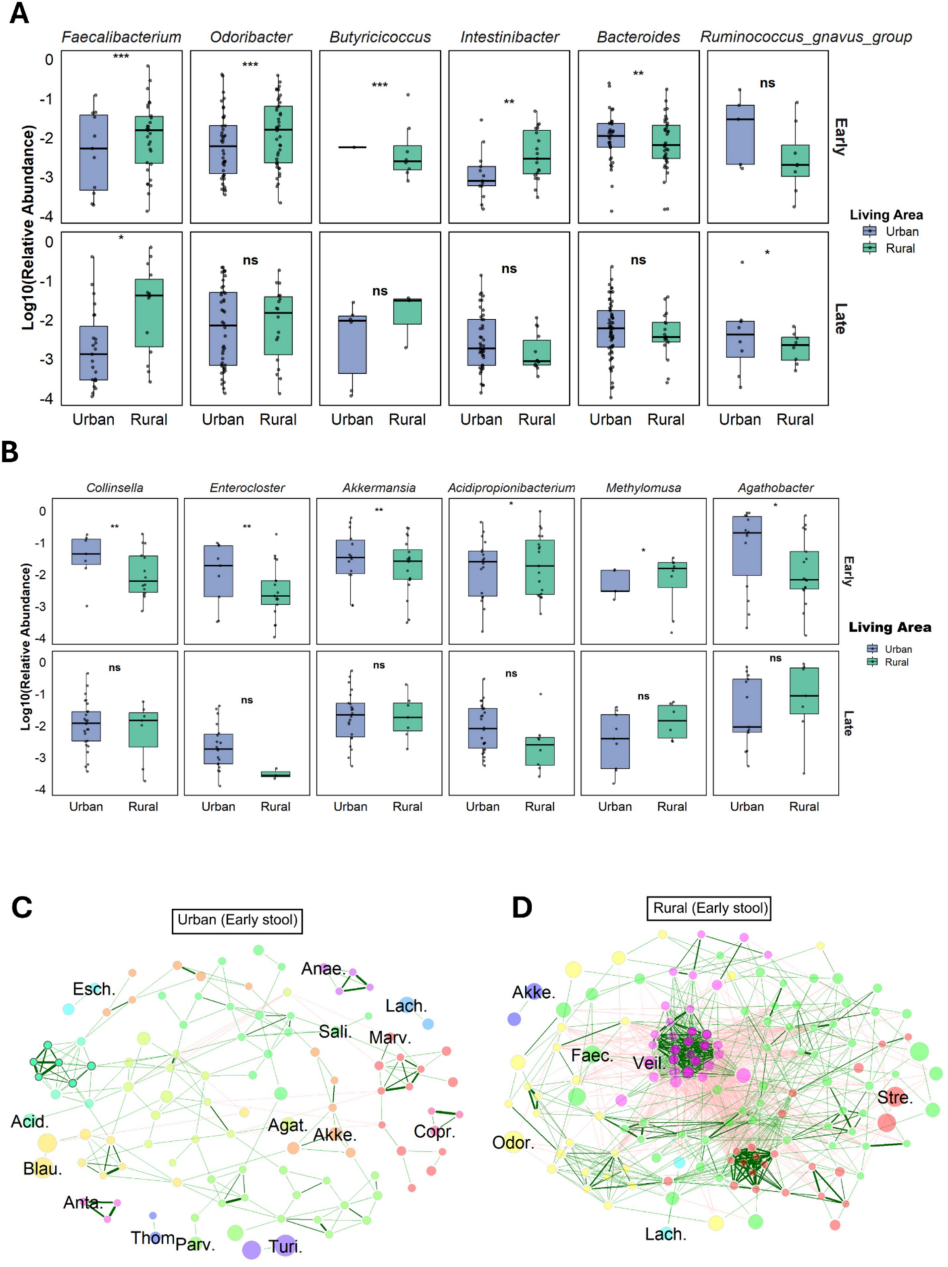
Effect of geographic location on taxonomic signatures and co-occurrence network. **(A,B)** Boxplots of log10-transformed relative abundance of selected genera between rural and urban areas at early and late stages. *p (FDR) < 0.05; **p (FDR) < 0.01; ***p (FDR) < 0.001; ns, not significant. Co-occurrence networks of infant gut bacterial genera in **(C)** Urban early, and **(D)** Rural early infant stool samples. These are the most influential bacteria in the network, based on eigenvector centrality. Node size represents abundance and reflects centrality, edge thickness represents correlation strength (green: positive, red: negative), abbreviate labels indicate the dominant genus within each cluster, with only the most representative taxa shown, and node colors denote different clusters. The network topology illustrates clustering and co-occurrence patterns. Early stool: 0–2 months; Late stool:6–12 months. Urban Early: Acid., *Acidipropionibacterium*; Agat., *Agathobacter*; Akke., *Akkermansia*; Anae., *Anaerofustis*; Anta., *Antarcticibacterium*; Blau., *Blautia*; Copr., *Coprococcus*; Esch., *Escherichia-Shigella*; Lach., *Lachnospiraceae*_UCG-008; Marv., *Marvinbryantia*; Parv., *Parvimonas*; Sali., *Salinispira*; Sega., *Segatella*; Thom., *Thomasclavelia*; Turi., *Turicibacter*. Rural Early: Akke., *Akkermansia*; Faec., *Faecalibacterium*; Lach., *Lachnospiraceae_NK4A136_group*; Odor., *Odoribacter*; Sega., *Segatella*; Stre., *Streptococcus*; Veil., *Veillonella*.

Relative abundance boxplots of phylum taxa in infant stool showed significantly higher abundances of *Bacillota* and *Bacteroidota*, *Planctomycetota*, *Actinomycetota*, *Pseudomonadota*, *Rhodothermota* and *Verrucimicrobiota* in urban infant early stool, and higher *Planctomycetota* were detected in urban infant late stool compared to corresponding rural infant stool samples (Supplementary Figure S2). The relative abundances of top 20 abundant genera in the stool of rural-living infants contained significantly higher levels of *Faecalibacterium*, *Odoribacter*, *Intestinibacter*, *Methylomusa*, and *Acidipropionibacterium* during their early stage. In contrast, infants living in urban areas had higher relative abundances of *Bacteroides*, *Agathobacter*, *Collinsella*, *Enterocloster*, and *Akkermansia* (Figures 4A,B). During later infancy, rural infants still exhibited significantly higher levels of *Faecalibacterium*, while that of *Ruminococcis_gnavus_group* was significantly lower than that in the urban infant group (Figure 4A). A group of less frequency fecal genus taxa had significant differences between regional groups and were displayed in Supplementary Figure S3. Significantly higher abundances of *Haemophilus*, *Coprococcus*, *Ruminococcus*, *Blaucia*, *Dorea*, *Steptococcus*, *Segatella*, *Parabacteroides*, and *UCG-002* were detected in early stool, and lower *Bifidobacteria* and *Acidipropionibacterium* were found in urban infant early stool compared to rural counterparts; higher *Howardella*, *Weissella*, *Dorea* and *UCG-002* were detected in late urban stool compared to late rural stool (Supplementary Figures S3A,B).

The co*-*occurrence network analysis showed that the early urban infancy gut microbiota was less cohesive with dominating hubs around 14 taxa, including *Akkermansia* and *Agathobacter* (Figure 4C). In contrast, early fecal networks were evidently more interconnected in rural infants, with more negative associations between taxa among the lower central genera hubs. *Faecalibacterium* and *Odoribacter* were notable hub taxa that we also detected as high frequency in rural early infancy stool (Figure 4D).

In the late infant stool of urban areas, the networks remained sparsely connected, but the taxa from early stool did not stay consistent except for *Akkermansia* (Supplementary Figure S4A). In rural samples, more negative associations were also observed in the late stage, without an increase in the number of dominant hub taxa. The presence of *Faecalibacterium* and *Odoribacter* indicated a more rigid network with reduced bacterial turnover, potentially reflecting an inhibitory effect of these taxa (Supplementary Figure S4B).

### 3.4 Geographic differences in infant fecal SCFA_S_ and relationship with gut microbiota

**Figure 5:**
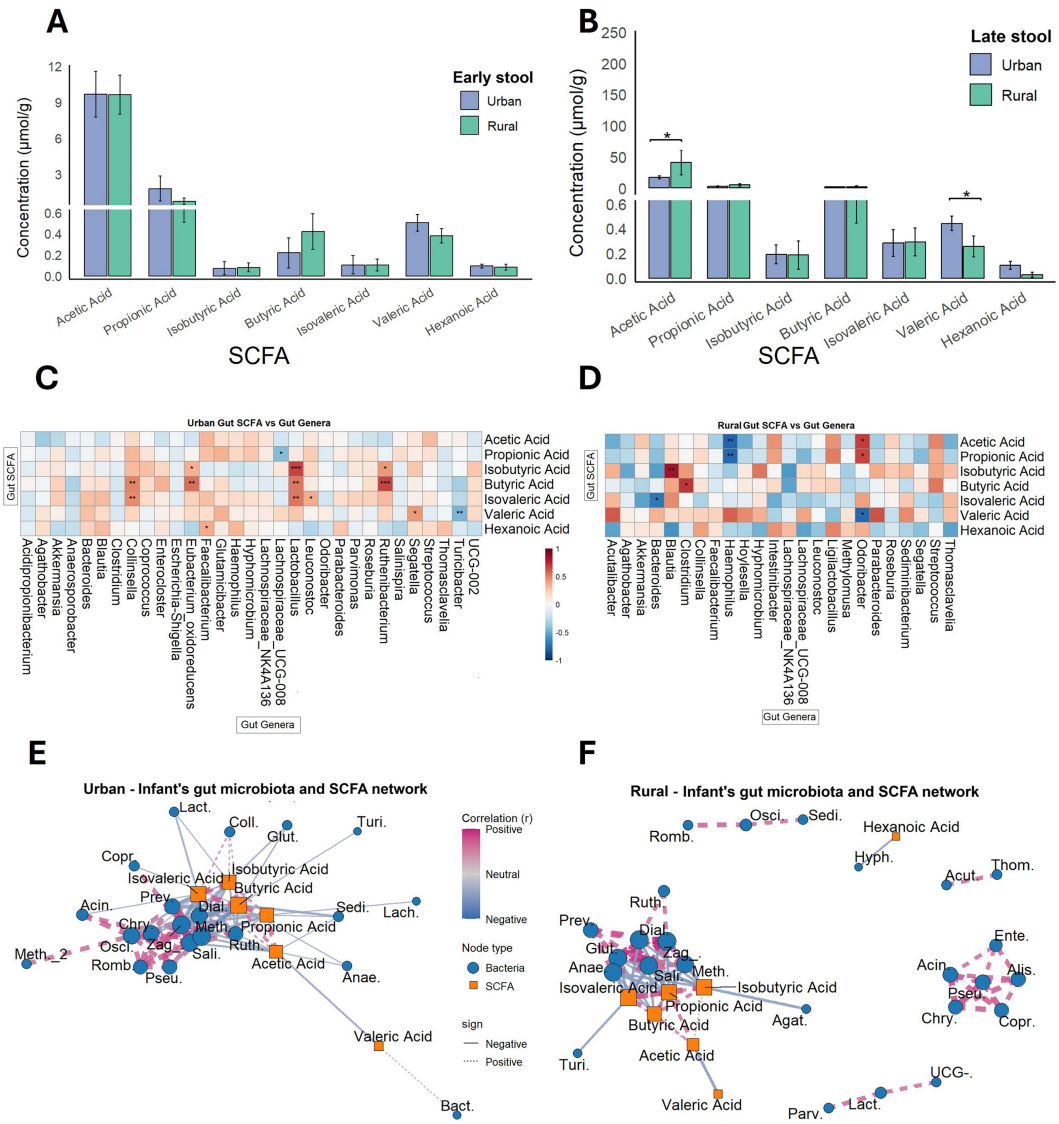
Effect of geographic location on short-chain fatty acid (SCFA) profiles in infant gut microbiota and Urban–Rural differences in shared milk-gut taxa. **(A,B)** Bar plots show normalized concentrations of SCFA in infant gut microbiota from urban and rural mothers during **(A)** early and **(B)** late stages. Asterisks denote significance (Wilcoxon test, *p (FDR) < 0.01). Heatmaps show Spearman correlations between gut bacterial genera and SCFAs in **(C)** urban and **(D)** rural late-stage infants. Asterisks indicate significant correlations *p (FDR) < 0.05; **p (FDR) < 0.01; ***p (FDR) < 0.001. Early stool: 0–2 months; Late stool: 6–12 months, respectively. Co-occurrence networks between infant gut microbiota in genera level and SCFAs in **(E)** urban, and **(F)** rural early infant stool samples. Acin., *Acinetobacter*; Akke., *Akkermansia*; Alis., *Alistipes*; Anae., *Anaerococcus*; Bact., *Bacteroides*; Blau., *Blautia*; Chry., *Chryseobacterium*; Clos., *Clostridium*; Copr., *Coprococcus*; Cory., *Corynebacterium*; Dech., *Dechloromonas*; Dial., *Dialister*; Eliz., *Elizabethkingia*; Glut., *Glutamicibacter*; Haem., *Haemophilus*; Lach., *Lachnospiraceae_UCG-008*; Lact., *Lactobacillus*; Leuc., *Leuconostoc*; Ligi., *Ligilactobacillus*; Odor., *Odoribacter*; Osci., *Oscillibacter*; Para., *Parabacteroides*; Pept., *Peptoniphilus*; Prev., *Prevotella*; Pseu., *Pseudomonas*; Romb., *Romboutsia*; Rose., *Roseburia*; Sedi., *Sediminibacterium*; Sega., *Segatella*; Stre., *Streptococcus*; UCG-., *UCG-002*; Zag_., *Zag_111*.

The abundance of fecal acetic acid in rural-living infants during later infancy was significantly higher than in urban-living infants. Meanwhile, the amount of fecal valeric acid during late infancy from urban-living infants was significantly higher than that in rural infants (Figures 5A,B). Levels of acetic acid and propionic acid positively correlated with *Odoribacter* and negatively correlated with *Haemophilus* in rural-living infants. Valeric acid showed a significant positive association with *Segatella* in urban infants (Figure 5C) but was negatively correlated with *Odoribacter* in urban infants and negatively associated with *Turicibacter* in rural infants (Figure 5D). Moreover, network analysis of the co-occurrence between SCFAs and bacteria revealed a negative association between valeric acid and acetic acid in both urban and rural infant stool samples. Additionally, *Bacteroides* showed a positive interaction with valeric acid in the urban group (Figures 5E,F).

### 3.5 Overlap between milk and infant gut microbiota varies by geographical residency and time

The urban group’s unique taxa in the infant gut microbiota grew over time (change from three to eight unique taxa), whereas the rural group mainly remained unchanged (Supplementary Figure S4C). We employed an UpSet plot to investigate microbial sharing between early and mature milk as well as early and late infant gut microbiota in urban and rural populations. Early milk microbiota (Early_Milk) and mature milk microbiota (Mature_Milk) shared twice as many taxa in urban samples (*n* = 41) compared to rural samples (*n* = 20) (Supplementary Figures S4D,E).

ASV sharing between matched milk–gut samples from both rural and urban populations were examined to determine possible microbial continuity and divergence between breast milk and infant stool. Overall, urban dyads shared a median of four ASVs (range: 0–11), whereas rural dyads shared a median of one ASV (range: 1–2) between mature milk and late infant gut microbiota (Supplementary Figures S4F,G). Considering this, urban dyads had 4 times more shared microbes between milk and infant gut compared to rural counterparts, which may represent that rural dyads exhibited a higher divergence between milk and gut microbiota. In contrast, urban dyads had less divergent and more continuity between milk and infant gut microbiota.

### 3.6 Interaction between breast milk and infant gut bacteria from rural and urban dyads

**Figure 6:**
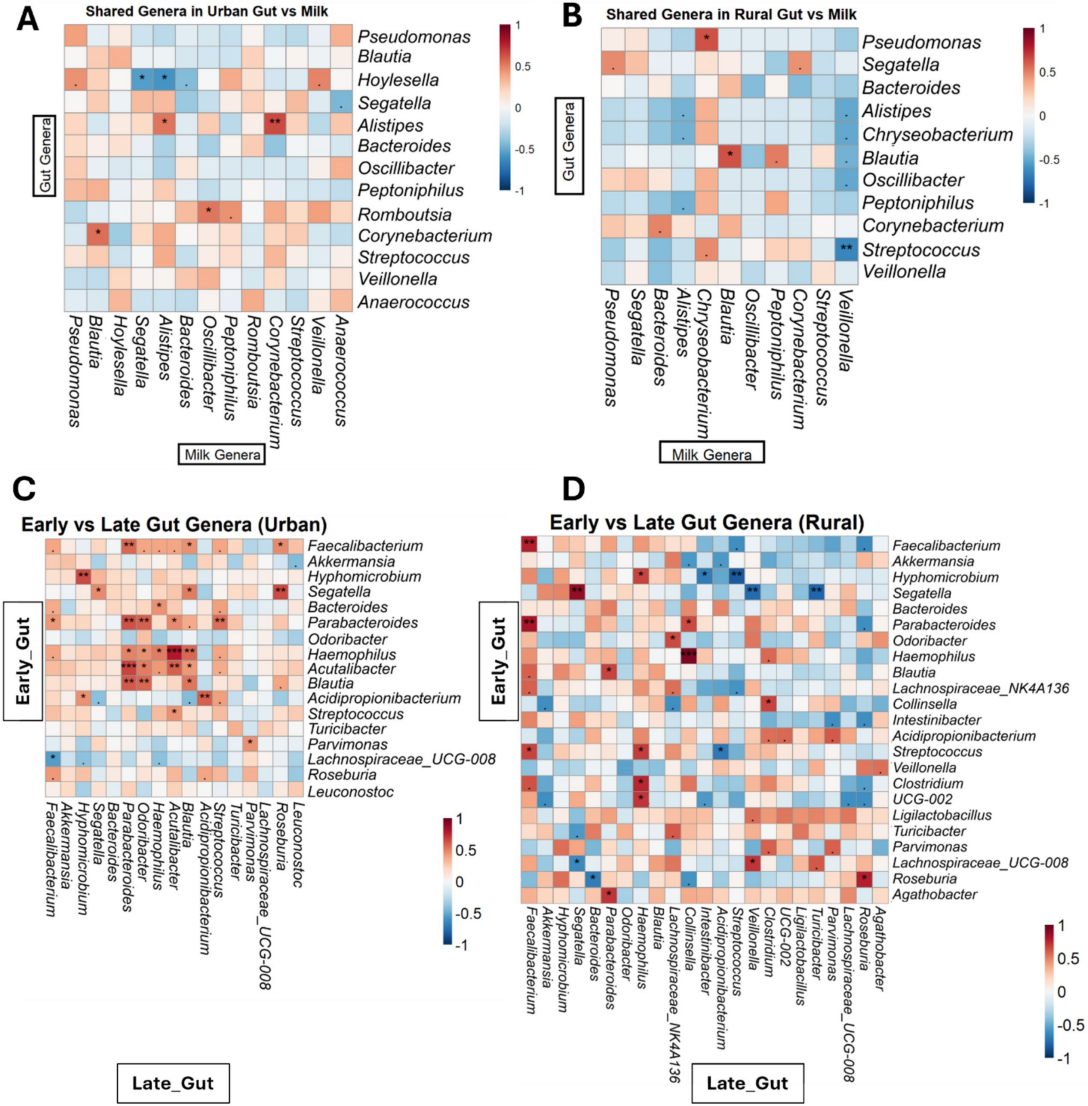
Shared taxa and correlations between milk and gut microbiota genera from urban and rural-living mothers and offspring. Heatmaps of genus-level correlations between milk and infant gut microbiota in **(A)** urban and **(B)** rural groups. **(C,D)** Heatmaps of correlations between infant gut microbiota in early and late infancy. Early_Gut, early infant gut microbiota (0–2 months); Late-Gut, late infant gut microbiota (6–12 months); Milk Genera, milk microbiota; Gut Genera, infant gut microbiota. Correlation strength is indicated by color gradient (blue: negative, red: positive), with values ranging from −1 to 1. Dot (.): *p* = 0.05; *p < 0.05; **p < 0.01; ***p < 0.001.

In urban dyads, the abundances of *Alistipes* in milk and infant stool were positively associated, indicating microbial potential vertical transmission (Figure 6A). Milk *Blautia* was positively linked to gut *Corynebacterium*, and milk *Corynebacterium* positively correlated with gut *Alistipes*, suggesting potential inter-genus promotion. Milk *Oscillibacter* and *Peptoniphilus* were positively associated with gut *Romboutsia*. In contrast, *Segatella* and *Alistipes* in milk negatively correlated with gut *Hoylesella*, implying possible inhibitory effects (Figure 6A). Rural milk and infant stool *Blautia* were positively correlated, suggesting that breastfeeding may accelerate milk-gut sharing of this genus of bacteria. Conversely, milk *Veillonella* displayed a strong inverse relationship with gut *Streptococcus* (*p* < 0.01) but weaker negative correlations with gut *Alistipes*, *Oscillibacter*, and *Blautia* (Figure 6B). In urban infants’ gut microbiota, early *Segatella* was positively associated with late *Blautia*, *Segetella*, and *Roseburia* (*p* < 0.05 or 0.01). Early *Parabacteroide*s positively correlated with late *Acutalibacter*, *Blautia*, and *Faecalibacterium* (*p* < 0.05 or 0.01, Figure 6C). In rural infants, early *Faecalibacterium*, *Segetella*, and *Roseburia* maintained positive associations with their late counterparts (Figure 6D; see Tables 2, 3 for a summary of major findings).

**Table 2:** Summary of microbial sharing, continuity, and interaction patterns between urban and rural areas.

Location	Milk → Gut shared taxa	Gut → Gut continuity (# of significant pairs)	Promote or inhibit	Dominant process
Urban	1 (*Alistipes → Alistipes*)	High (strong self-continuities: *Hyphomicrobium*, *Parabacteroides*, *Segatella*, *Blautia*, *Odoribacter*)	Mainly promote (multiple positive intra- and inter-genus associations)	Gut continuity & promotive succession
Rural	1 (*Blautia → Blautia*)	Moderate (self-continuity in *Faecalibacterium*, *Segatella*, *Roseburia;* mixed interactions)	More mixed, with potent inhibition (e.g., *Veillonella* inhibiting multiple genera)	Mixed shared taxa with ecological filtering

**Table 3:** Overview of microbial and metabolic characteristics in urban versus rural infants and their potential effects on diabetes risk.

Feature	Rural infants	Urban infants	Hypothesis-based mechanisms from literature
Diabetes-associated taxa	*Faecalibacterium*, *Blautia*, *Odoribacter*	*Akkermansia*, *Alistipes*, *Bacteroides*, *Veillonella*, *Acidipropionibacterium*, *Ruminococcaceae*	*Urban*: Some T2D risk with *Veillonella*, but outweighed by protective taxa against T2D such as *Akkermansia*, *Alistipes*, *Bacteroides*, *Acidipropionibacterium*, and *Ruminococcaceae.* *Rural*: *Faecalibacterium* is negatively associated with T2D, but *Odorobacter* and *Blautia* are recognized as markers for patients with T2D.
Main SCFAs	Higher acetic acid (in gut), isobutyric acid (in milk)	Higher valeric acid (in gut)	*Rural*: Acetic acid protective; *Urban*: Valeric acid supports insulin sensitivity.
Succession pattern	Early maturity but mixed, unstable later	Structured, promotive, cohesive	*Rural*: Dynamic, may predispose to dysregulation with higher risk for T2D; *Urban*: Cohesive, supports resilience to diabetes.
Microbial continuity	Lower overlap between milk and gut, lower gut–gut	Higher overlap between milk and gut, high gut–gut continuity	*Urban*: Higher continuity supports gut maturation and protective effects for diabetes.

T2D, Type 2 diabetic.

### 3.7 Associations between infant fecal SCFAs and breast milk microbiota or SCFAs from rural and urban dyads

**Figure 7:**
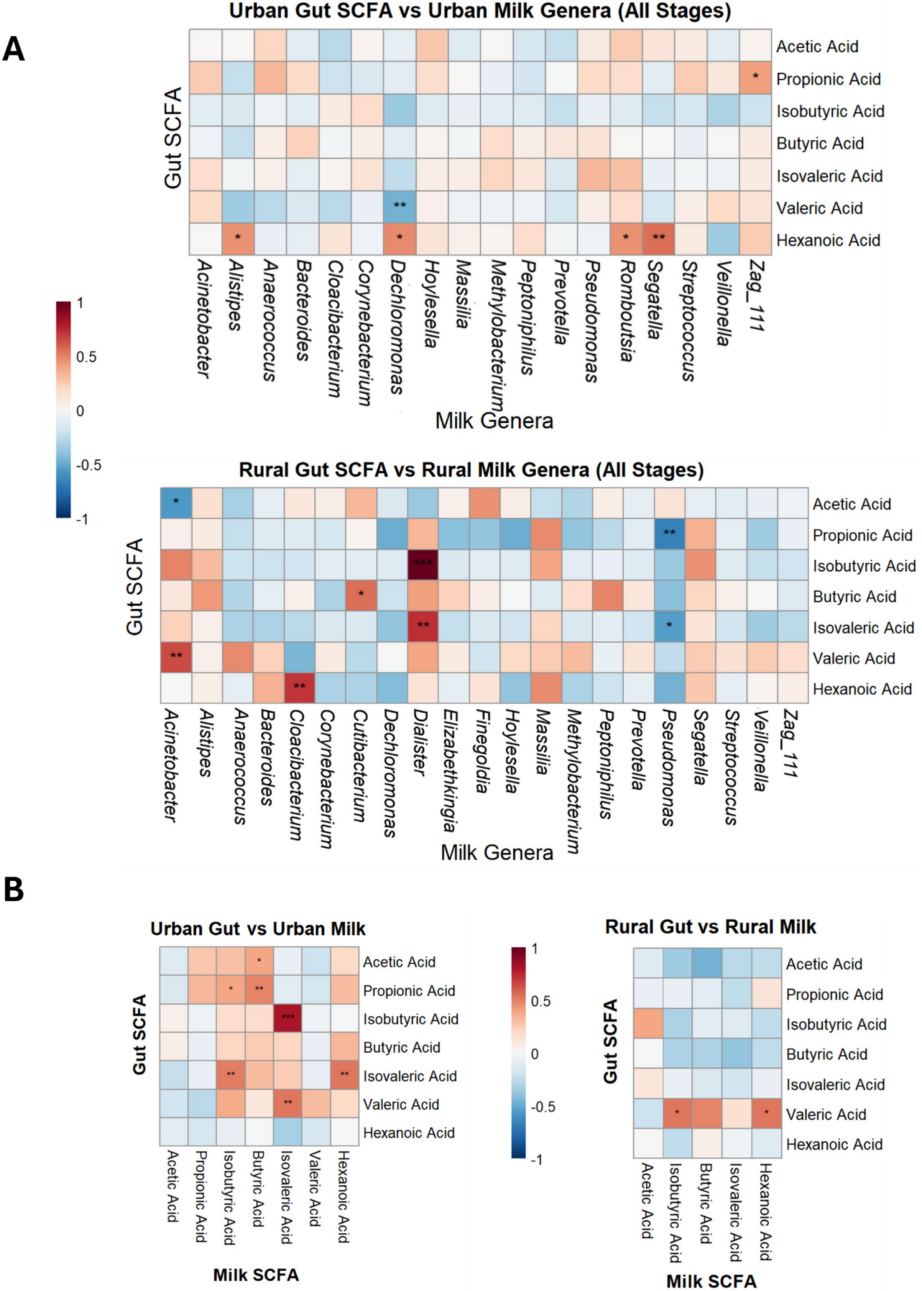
Associations between infant gut short-chain fatty acids (SCFAs) and milk bacteria and SCFAs. **(A)** Heatmaps showing Spearman correlations between milk bacterial genera and infant fecal SCFAs in urban (upper) and rural (bottom) populations (all stages combined). Only genera with at least 1% relative abundance in one sample and present in ≥30% of samples were included. **(B)** Heatmaps illustrating correlations between gut SCFAs and milk SCFAs in urban (left) and rural (right) infants. Significant associations after FDR correction are marked with asterisks. Early stool: 0–2 months; Late stool: 6–12 months. *p (FDR) < 0.05; **p (FDR) < 0.01; ***p (FDR) < 0.001.

Rural and urban groups showed different patterns in associations between gut SCFAs and milk genera microbiota, and SCFAs between milk and gut in rural or urban settings (Figures 7A,B). Milk *Segatella*, *Romboutsia*, and *Alistipes* exhibited positive correlations with infant fecal hexanoic acid in urban areas. Milk *Dechloromonas* negatively correlated with valeric acids and positively associated with hexanoic acid in urban infants. Milk *Zag_111* was positively correlated with infant fecal propionic acids (*p* < 0.05–0.01, Figure 7A, upper). In rural dyads, significantly positive associations between infant gut SCFAs and milk genera, including isobutyric and isovaleric acids with *Dialister*, hexanoic acid with *Cloacibacterium*, valeric acid with *Acinetobacter*, and butyric acid with *Cutibacterium* were found (*p* < 0.05–0.001). Negative correlation between acetic acid and *Acinetobacter*, and between *Pseudomonas* and propionic acid or isovaleric acid, was observed in rural dyads (*p* < 0.05–0.01, Figure 7A, bottom).

None of the SCFAs in breast milk and infant stool in urban or rural dyads was positively associated with itself. Positive associations were detected between milk isovaleric acid and infant stool isobutyric acid, between milk isobutyric acid and stool isovaleric acid or propionic acid, milk hexanoic acid and stool isovaleric acid, and between milk butyric acid and infant stool acetic or propionic acids in urban dyads (Figure 7B left). Positive correlations between stool valeric acid and milk isobutyric acid or hexanoic acid were detected in rural dyads (Figure 7B, right).

## 4 Discussion

Previous research has highlighted the dynamic nature of breast milk microbiota and its role in shaping infant gut colonization (Moossavi et al., 2019). Limited studies have simultaneously examined how geographic residency affects maternal milk and infant gut microbiota and their metabolites across different stages of infancy or nursing. To address this gap, the present study compared the microbiota and SCFA profiles in breast milk within the first month postpartum and in stool collected during the first year of dyads living in urban versus rural areas in Manitoba, Canada. Distinct differences in microbial diversity, composition, maturity, co-occurrence networks, SCFA levels, and relationships between bacteria and SCFAs were detected between urban and rural dyads.

Breast milk from rural mothers contained higher levels of specific genera, such as *Dechloromonas* and *Segatella*, while urban mature milk had more *Veillonella* and *Alistipes*. *Dechloromonas* is a genus commonly abundant in individuals with T2D (Yin et al., 2020). *Segatella* is a prevalent genus of bacterium, especially in non-Western populations. *Segatella copri* is a species of *Segatella* linked to insulin resistance (Pedersen et al., 2016; Xiao et al., 2024). Both increased and decreased levels of *Veillonella* have been observed in T2D patients, highlighting the complex and context-dependent relationship between this bacterium and T2D (Letchumanan et al., 2022). *Alistipes*, particularly *Alistipes indistinctus*, reduced insulin resistance and blood sugar in animal models (Zhang, 2024). The rate of breastfeeding initiation in urban-living infants in Manitoba was significantly higher than in rural-living infants (Hui et al., 2025). Breastfeeding initiation was associated with lower postpartum diabetes in mothers and youth-onset T2D in offspring in Manitoba (Martens et al., 2016). Considering the previous findings and the results of the present study, the differences in milk microbiota composition and breastfeeding initiation between rural and urban-living dyads suggest a hypothetical association with the higher risk of diabetes in rural-living people in Canada as previously described (Spark Conference, 2025).

We observed both promotive and inhibitory effects of milk microbiota in shaping the gut colonization in rural and urban infants. In urban infants, positive intra- and inter-genus correlations between milk and infant gut microbiota suggest possible coordinated microbial succession, particularly *Alistipes*, *Corynebacterium*, and *Romboutsia*, which are linked to the development of metabolic and immune systems (Arrieta et al., 2014; Milani et al., 2017). In contrast, rural infants showed both promotive (e.g., *Blautia* to *Blautia*) and inhibitory effects, notably strong suppression by milk *Veillonella* against gut genera such as *Streptococcus*. These inhibitory interactions may result from competitive exclusion or ecological filtering shaped by higher microbial diversity in rural environments (Subramanian et al., 2014; Yatsunenko et al., 2012). Overlapping ASVs in milk and stool samples suggest sharing microbial between breast milk to the infant’s gut. Rural dyads with lower ASV sharing at both early and mature stages highlight a greater divergence in the two microbiota pools. Notably, milk and stool pools among urban dyads showed a higher ASV sharing during early infancy, suggesting early-life microbiota overlap and less divergence. These geographic differences in ASV overlap imply that infant gut colonization depends more on environmental and host factors than on direct milk transfer (Bogaert et al., 2023). According to a previous report, urban milk samples exhibited more microbial overlap between early and mature milk compared to rural samples, suggesting a more consistently preserved milk microbiome over time (Pannaraj et al., 2017). A less divergent and more preserved milk microbiome may provide a relatively greater protective effect against metabolic diseases in urban infants than rural counterparts.

The present study performed a comparative evaluation to determine whether specific signatures in the infant gut microbiota could be linked to a protective ecosystem for metabolic disorders. A large battery of gut bacteria or microbial patterns has been described with association with T2D or the glucose-lowering effect of metformin (Balvers et al., 2021; González et al., 2025; Gravdal et al., 2023; Zhao et al., 2019). Our observations showed that rural infants had a greater gut microbiota *α*-diversity and maturity within the first two months of life compared to urban infants. This pattern indicates that early microbial diversity and maturity may be influenced by rural environmental factors, such as water quality, soil, animal contact, reduced sanitation, and fewer medical interventions that promote early colonization and diversity, and maternal dietary or physical activity habits, as previously described (Dominguez-Bello et al., 2010; Subramanian et al., 2014; Yatsunenko et al., 2012). Conversely, urban infants showed a delayed increase in gut microbiota diversity, consistent with reports from Morandini and colleagues (Morandini et al., 2023). The higher microbial maturity and succession patterns observed in guts of urban infants 6–12 months of age may represent a relatively lower risk of metabolic disorders and inflammatory responses compared to rural infants as previously described (Li and Li, 2023). These findings imply that infant gut microbial diversity and maturation could be influenced, in part, by residential location in an age-dependent manner as previously described (Morandini et al., 2023; Pace et al., 2021).

In addition, urban infant stool samples showed a higher relative abundance of *Akkermansia*, *Acidipropionibacterium*, *Bacteroides*, and *Ruminococcaceae*, compared to rural infants, some of which have been linked to urban environments and immune responses (Subramanian et al., 2014). *Akkermansia* is recognized for its potential role in improving glucose metabolism (Li et al., 2023; Zhang et al., 2025). A prior study suggests that *Acidipropionibacterium* may aid in improving metabolism by producing butyrate and activating GPR41, a receptor for SCFAs plays critical roles in upregulation of glucose uptake and insulin sensitivity (Miyamoto et al., 2025). *Bacteroides* and *Ruminococcaceae* were negatively associated with T2D risk (Ejtahed et al., 2020; Gravdal et al., 2023; Li and Li, 2023). Meanwhile, *Alistipes* showed overlap between milk and the infant gut in urban, but not in rural, dyads, and persisted from early to late infancy. The combination of findings from the present and previous studies suggests that urban infants’ guts contain more hypnotically protective bacteria profiles against diabetes, and breastfeeding may contribute to the healthier microbiota in urban infants’ guts.

A distinct composition of bacteria was detected in rural-living infants. The abundance of *Odoribacter*, a genus taxon associated with T2D patients (Hendricks et al., 2023), was higher in rural-living infants compared to urban-living infants. Sharing of *Blautia* between milk and infant gut in rural-living infants may increase the risk of diabetes as previously indicated (Gravdal et al., 2023; Qin et al., 2012), although certain species of *Blautia*, such as *Blautia wexlarae*, may play a protective role against obesity (Hosomi et al., 2022). Relative lower abundances of fecal *Akkermansia*, *Acidipropionibacterium*, *Bacteroides*, and *Ruminococcaceae* in rural infants potentially increased risk of T2D compared to urban infants (Letchumanan et al., 2022; Semo et al., 2024; Zhou et al., 2024). Higher levels of certain bacteria in rural infants may result from exposure to different environmental variables, such as gradual lifestyle and dietary shifts toward more urbanize dietary habits which can change in gut microbiota composition (Lu et al., 2021; Oduaran et al., 2020). One of these bacterial change we observed in abundances of *Faecalibacterium*, which is well known for its anti-inflammatory and butyrogenic properties, was more abundant in rural milk and stool samples. *Faecalibacterium* has also been linked to a lower risk of T2D and chronic inflammation (Lopez-Siles et al., 2017; Qin et al., 2012; Sokol et al., 2008). The combination of the findings in the profile of gut microbiota associated with rural infants suggests more complicated relationships between diabetes susceptibility and gut bacteria in rural infants.

Since SCFAs influence host metabolism, intestinal permeability, and immune development, these changes are both ecologically and clinically important. In urban late infancy, higher concentrations of fecal valeric acid were detected compared to those of rural infants. Velaric acid increases insulin responsiveness and basal glucose uptake, mainly through GPR41 signaling, in insulin-sensitive tissues (Han et al., 2014). Valeric acid has also been linked to immune signaling and energy balance (Ríos-Covián et al., 2016). Valeric acid is produced by anaerobic bacteria, including some types of *Segatella* (Xiao et al., 2024). Positive and significant correlation between valeric acid and *Segatella* was detected in urban, but not rural, infant stool. A moderate, but significantly, higher abundances of acetic acid were detected in late stool from urban infants compared to rural infants. Although the role of acetic acid in metabolism remains uncertain, the results of a recent study suggest that higher fecal acetic acids in infants is associated with breastfeeding and *Bifidobacteria*-mediated fermentation (Aatsinki et al., 2024). The findings of the present study indicated that rural and urban infants had distinct profiles of gut -derived SCFAs, which are influenced by gut microbiota, feeding and geographic residence as previously described (Lu et al., 2021; Oduaran et al., 2020).

Mature milk from rural areas had higher levels of isobutyric acid, a branched SCFA (BSCFA). The relationship between BSCFAs, isovaleric and isobutyric acids, and diabetes remains unclear (Wang S. et al., 2025). BSCFA improved insulin-induced glucose uptake and inhibited lipolysis in cultured adipocyte (Heimann et al., 2016). High levels of circulatory BSCFAs were associated with low odds of dysglycemia and improved glucose homeostasis in human subjects (Aslamy et al., 2024). The relatively higher levels of isobutyric acid in rural mothers’ milk may result from more active microbial fermentation of BCAAs or increased intake of protein-rich foods in the rural-living women during lactation. The present study demonstrated that the interactions of *Romboutsia* and *Corynebacterium* with isobutyric and hexanoic acids in rural milk samples, which suggest shared ecological niches promoting the generation of branched SCFAs or cooperative fermentative metabolism. Similar associations have been reported previously, *Corynebacterium glutamicum* supplementation raised isobutyric acid concentrations in murine models. *Romboutsia* abundance was linked to isobutyric acid levels in human gut samples, and hexanoic acid exhibited positive correlations with other SCFAs, including isobutyric acid (Chen et al., 2022; Liu et al., 2025). The finding suggests that breastfeeding by rural-living mothers may introduce more isobutyric acid to the gut of rural infants, which may bring benefit on the metabolic health to their offspring through a pathway distinguishable from that in urban dyads.

Bacteria co-occurrence network analysis revealed notable differences in milk and gut microbial organization between urban and rural dyads. Urban gut microbiota networks were more dispersed and showed high turnover of dominant taxa, while rural infant networks, dominated by taxa such as *Odoribacter* and *Faecalibacterium*, remained consistent across early and late stages. Notably, rural networks exhibited a higher proportion of negative associations, indicating regulatory interactions that may decrease ecological stability by preventing overgrowth of individual taxa. These results suggest that environmental context influences not only microbial composition but also the structural resilience of microbial communities. According to ecological principles, stable and well-connected networks are associated with stability and functional redundancy, whereas active networks may represent unstable or disruptive network (Coyte et al., 2015; Sun et al., 2023). A stable, well-structured early gut microbiota network, as observed in urban infants, has been associated with improved immune tolerance and a reduced risk of metabolic diseases, including diabetes (Vatanen et al., 2018). Conversely, disrupted or less coherent microbiota development, as seen in rural infants’ bacteria network, could increase susceptibility to immune dysregulation and chronic illnesses (Kostic et al., 2015).

## 5 Strengths and limitations

The main strength of this study is that stool and breast milk samples were collected from rural and urban dyads during the earliest period after delivery, a critical time for the initial development of gut microbiota and parallel assessment of SCFAs, which is not well documented in the literature among those populations. However, the study has some limitations, such as a smaller sample size, due to the requirement of longitude sample collections of milk and infant stool samples, which could lower the statistical power for microbial network comparisons and subgroup analyses. Besides, the present study has not collected simultaneous data on maternal dietary intake simultaneous with sample collection and some relevant maternal and infant growth variables, partially due to pandemic-associated social isolation and travel restriction, and lack of data on the development of metabolic diseases in participating mothers or offspring after delivery.

## 6 Conclusion

Maternal milk, infant gut microbiota and SCFA patterns during the first year of infancy were substantially influenced by rural versus urban residency in this prospective cohort. Compared to rural milk, urban milk bacteria exhibited more positive cohesive links with infant gut microbiota and higher overlap between early and mature milk bacteria. While rural infants’ gut microbiota gained more diversity in the first 2 months of infancy, but less consistent and more inhibitory interactions associated with delayed gut microbiota maturation were detected in their late infancy. The high-frequency taxa in breast milk and infant stool were also distinguishable between rural versus urban dyads. Urban milk contained more *Veillonella* and *Alistipes* compared to rural milk, which may contribute to microbial development for infants. Rural milk contained higher levels of isobutyric acid than urban milk, which may bring immune benefits to infants. Therefore, breastfeeding infants in both urban and rural regions may bring distinctive positive effect to infants. Urban infant stool contained more *Akkermansia*, *Acidipropionibacterium*, *Bacteroides*, *Ruminococcaceae*, and higher levels of valeric acids compared to rural infants. Rural infant stool had higher abundance of *Blautia* and higher level of acetic acid compared to urban infants. Milk and fecal microbiota composition and SCFA production are expected to be influenced by graphical, environmental and maternal diet associated with residential locations. The findings of the present study suggest that breastfeeding, the staple food of infants, may benefit the development and maturation of gut microbiota in infants living in both urban and rural regions in distinguishable pathways. The influence of breast milk and infant gut microbiota and SCFAs composition on chronic metabolic diseases may require to be further assessed in subsequent large-scale prospective studies accompanied with maternal and infant dietary intake information.

## Data Availability

Raw data analyzed in this study deposited at Figshare at https://doi.org/10.6084/m9.figshare.30653987.v3. The raw sequencing data deposited in the NCBI accession number PRJNA1375700.
