# Supplementary material for: Distinct patterns and interactions of microbiota and short-chain fatty acids in breast milk and infant gut from rural and urban dyads
Source: Front Microbiol. 2026 May 29;17:1814630. doi: 10.3389/fmicb.2026.1814630 (PMC13260080; doi:10.3389/fmicb.2026.1814630)
Supplement: Supplementary file 1 [file Table_1.DOCX]

**Distinct patterns and interactions of microbiota and short-chain fatty acids in breast milk and infant gut from rural and urban dyads**

Saeid Khakisahneh ^1^, Eunseo Lee^1^, Amy Hui^1^, Ruozhi Zhao^1,2^, Fei Huang^2^,

Xue-Ying Zhang^3,4^, Garry X. Shen^1, 2*^.

**Supplementary Figures and Tables:**

**Table S1.** Characteristics of milk samples

| **Category** | **Subcategory** | | **Quality Filter stage** | |
| --- | --- | --- | --- | --- |
|  |  |  | **Before**  **(n)** | **After**  **(n)** |
| Location | Urban | | 100 | 84 |
|  | Rural | | 50 | 34 |
| Milk stage | Urban | Early | 67 | 55 |
|  |  | Late | 33 | 29 |
|  | Rural | Early | 37 | 23 |
|  |  | Late | 13 | 11 |
| Delivery | Urban | Vaginal | 69 | 58 |
|  |  | C_section | 31 | 26 |
|  | Rural | Vaginal | 36 | 26 |
|  |  | C_section | 14 | 8 |
| GDM | Urban | GDM | 18 | 16 |
|  |  | Non-GDM | 82 | 68 |
|  | Rural | GDM | 3 | 3 |
|  |  | Non-GDM | 47 | 31 |
| Total |  | | 150 | 118 |

GDM, Gestational Diabetes Mellitus.

**Table S2.** Characteristics of Stool Samples

| **Category** | **Subcategory** | | **Quality Filter stage** | |
| --- | --- | --- | --- | --- |
|  |  |  | **Before**  **(n)** | **After**  **(n)** |
| Location | Urban | | 100 | 99 |
|  | Rural | | 46 | 45 |
| Milk stage | Urban | Early | 61 | 60 |
|  |  | Late | 39 | 39 |
|  | Rural | Early | 35 | 34 |
|  |  | Late | 11 | 11 |
| Delivery | Urban | Vaginal | 85 | 84 |
|  |  | C-section | 15 | 15 |
|  | Rural | Vaginal | 37 | 36 |
|  |  | C-section | 9 | 9 |
| Feeding mood | Urban | Breastfeed | 77 | 76 |
|  |  | Formula feed | 4 | 4 |
|  |  | Mixed feed | 19 | 19 |
|  | Rural | Breastfeed | 29 | 28 |
|  |  | Formula feed | 9 | 9 |
|  |  | Mixed feed | 8 | 8 |
| GDM | Urban | GDM | 23 | 22 |
|  |  | Non-GDM | 77 | 77 |
|  | Rural | GDM | 4 | 4 |
|  |  | Non-GDM | 42 | 41 |
| Total |  | | 146 | 144 |


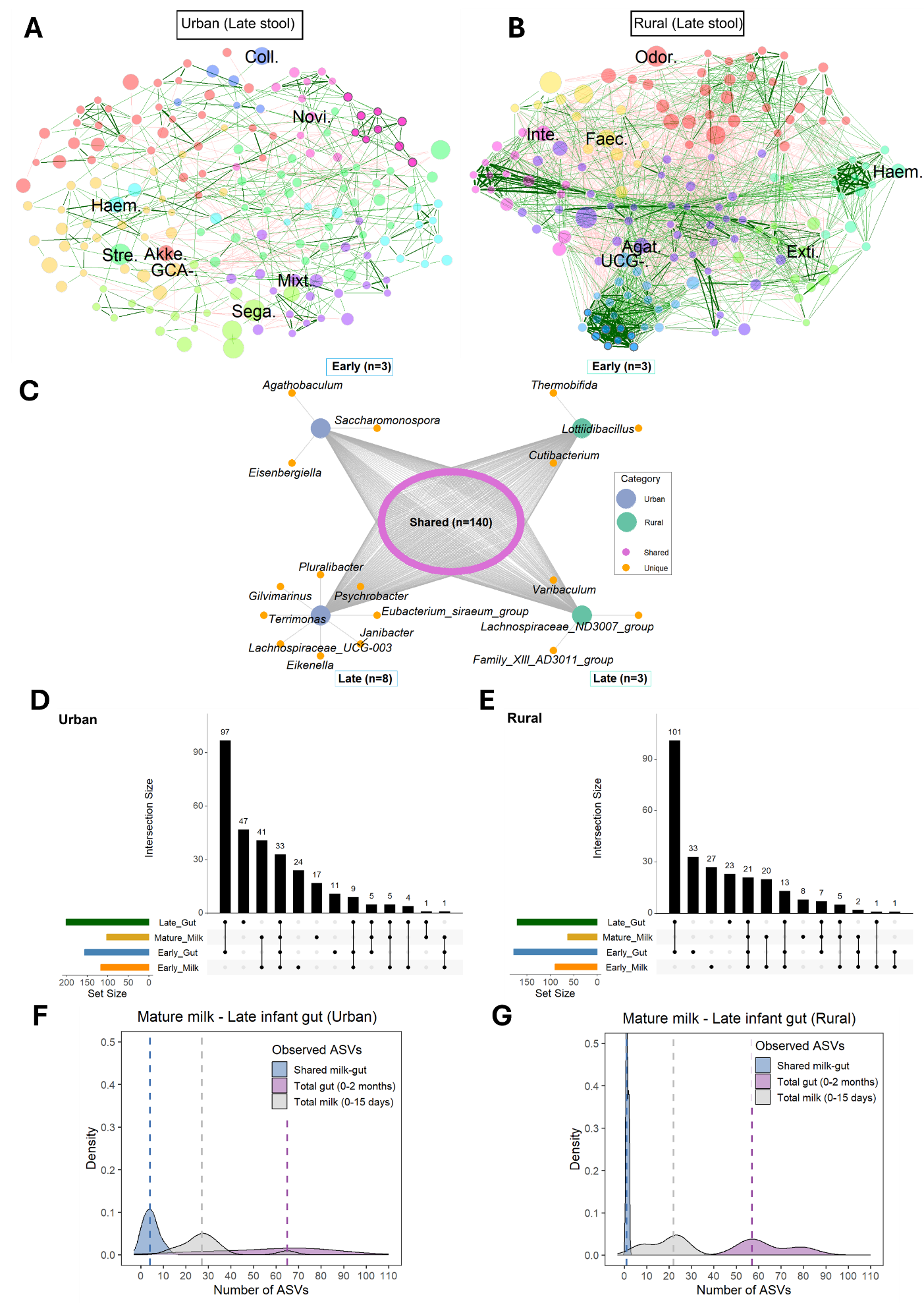


**Fig. S1. Infant gut microbial co-occurrence and overlap between urban and rural areas at timepoints.**

(A-B) Co-occurrence networks of infant gut bacterial genera in (A) Urban early and (B) Rural early infant stool samples. These are the most influential bacteria in the network, based on eigenvector centrality. Node size represents abundance and reflects centrality, edge thickness represents correlation strength (green: positive, red: negative), abbreviate labels indicate the dominant genus within each cluster, with only the most representative taxa shown, and node colors denote different clusters. The network topology illustrates clustering and co-occurrence patterns. (C) Bipartite network showing shared ASVs (purple, center) and group-specific ASVs (orange) across Early (0–2 months) and Late (6–12 months) stool in urban and rural groups. (D -E) UpSet plots showing shared bacterial genera between early and mature milk and infant gut microbiota across early and late stages in (D) urban and (E) rural infants. Numbers above bars indicate the count of shared bacterial genera in each intersection, out of the total detected genera in each set (Late_Gut: ~200, Mature_Milk: ~100, Early_Gut: ~90, Early_Milk: ~70), as shown by the horizontal bars on the left. Late-Gut, late infant gut microbiota; Early_Gut, early infant gut microbiota; Early_Milk, early milk microbiota; Mature_Milk, Mature milk microbiota. Early milk: 0–14 days; Mature milk: 15–30 days; Early stool: 0–2 months; Late stool: 6–12 months. (F–G) Density plots showing distributions for the number of amplicon sequence variants (ASVs) shared between mature milk and late infant stool of mother-infant dyads, and the total number of ASVs per milk and infant stool sample in urban (F) and rural (G). Medians are shown as dotted lines. The y-axis shows density, with higher density indicating more dyads with a given value. Network abbreviations: **Urban Late**: Agat., Agathobacter; Akke., Akkermansia; Coll., Collinsella; GCA-., GCA-900066575; Haem., Haemophilus; Lach., Lachnospiraceae_UC008; Mixt., Mixta; Novi., Novibacillus; Sega., Segatella; Stre., Streptococcus. **Rural Late**: Agat., Agathobacter; Exti., Extibacter; Faec., Faecalibacterium; Haem., Haemophilus; Inte., Intestinibacter; Odor., Odoribacter; Sega., Segatella; Stre., Streptococcus; UC., UC002.
